# Age-adjusted and Expanded Lactate Thresholds as Predictors of All-Cause Mortality in the Emergency Department

**DOI:** 10.5811/westjem.2020.5.46811

**Published:** 2020-08-20

**Authors:** Chad M. Cannon, Ross T. Miller, Krista L. Grow, Seth Purcell, Niaman Nazir

**Affiliations:** * University of Kansas Medical Center, Department of Emergency Medicine, Kansas City, Kansas; † University of Kansas Medical Center, Department of Preventative Medicine and Public Health, Kansas City, Kansas

## Abstract

**Introduction:**

While numerous studies have found emergency department (ED) lactate levels to be associated with increased in-hospital mortality, little information is available on the role age plays in this association. This study investigates whether age is a necessary variable to consider when using lactate levels as a marker of prognosis and a guide for management decisions in the ED.

**Methods:**

This was a retrospective cohort study in an urban, tertiary-care teaching hospital. A total of 13,506 lactate levels were obtained over a 4.5-year period. All adult patients who had a lactate level obtained by the treating provider in the ED were screened for inclusion. The main outcome measure was in-hospital mortality using age-adjusted cohorts and expanded lactate thresholds with secondary outcomes comparing mortality based on the primary clinical impression.

**Results:**

Of the 8796 patients in this analysis, there were 474 (5.4%) deaths. Mortality rates increased with both increasing lactate levels and increasing age. For all ages, mortality rates increased from 2.8% in the less than 2.0 millimoles per liter (mmol/L) lactate level, to 5.6% in the 2.0–2.9 mmol/L lactate level, to 8.0% in the 3.0–3.9 mmol/L lactate level, to 13.9% in the 4.0–4.9 mmol/L lactate level, to 13.7% in the 5.0–5.9 mmol/L lactate level, and to 39.1% in the 6.0 mmol/L or greater lactate level (p <0.0001). Survivors, regardless of age, had a mean lactate level <2.0 whereas non-survivors had mean lactate levels of 6.5, 4.5, and 3.7 mmol/L for age cohorts 18–39, 40–64, and ≥ 65 years, respectively.

**Conclusion:**

Our findings suggest that although lactate levels can be used as a prognostic tool to risk stratify ED patients, the traditional lactate level thresholds may need to be adjusted to account for varying risk based on age and clinical impressions.

## INTRODUCTION

Lactate has been studied as a marker of critical illness for over a half century.^1^ Lactate levels can be used as a surrogate of tissue hypoperfusion in critically ill patients presenting to the emergency department (ED). Lactate production and metabolism are critical to the ability of the body to respond to metabolic stressors and varying shock states.^2^ However, lactate may also be elevated due to varying conditions in the absence of tissue hypoxia through a variety of mechanisms.^3^,^4^ Lactate levels can readily be obtained and used to identify patients at high risk of death, even prior to the development of hemodynamic instability. Poor organ perfusion, if not reversed, ultimately leads to organ dysfunction and failure, shock, and potentially death.

The use of lactate levels has been shown to be a predictor of prognosis in diverse populations of critically ill patients ranging from trauma to septic shock.^5^–^10^ In the last decade, there has been increased use and evaluation of lactate in the ED and these studies demonstrate that elevated lactate levels are associated with increased mortality.^11^–^14^ Prior studies have already demonstrated the utility of lactate levels to predict mortality in patients admitted to the hospital who presented from the ED with infection^11^,^15^,^16^ and severe sepsis,^17^,^18^ as well as trauma.^19^

With an increasing focus on guidelines and quality performance measures, initiatives such as the Surviving Sepsis Campaign, the Centers for Medicare and Medicaid Services (CMS) core measures, the Medicare Access and CHIP Reauthorization Act of 2015 and the Merit Based Incentive Payments System quality measures have all incorporated the measurement of serum lactate levels into their most current guidelines, likely contributing to the increasing the number of lactate levels being ordered in the ED.^20^ Since the beginning of early goal-directed therapy (EGDT) lactate levels have been increasingly used as diagnostic, therapeutic, and prognostic markers in ED patients.^13^,^21^ As there are numerous causes for an elevated lactate, it is important for emergency physicians to consider both sepsis and alternative diagnoses, because the prognostic value of lactate can vary depending on the underlying cause.^22^ While lactate has been shown to be sensitive for occult sepsis, the current literature supports the notion that while it is highly sensitive, it is not specific.^23^,^35^

Historically, the same lactate stratification levels have been used regardless of patient age or disease state: low levels, < 2.0 millimoles per liter (mmol/L); intermediate levels, 2.0–3.9 mmol/L; and high levels, ≥ 4.0 mmol/L.^2^ Despite this cutoff of ≥ 4.0 mmol/L being used in models such as EGDT^21^ and CMS criteria for septic shock,^24^ the intermediate lactate level has also proven to be a high-risk group, as previously noted by Mikkelsen and Howell.^15^,^17^ In addition to investigating expansion of lactate threshold levels, adjusting these lactate thresholds for age may also contribute to additional discrimination. Portal et al showed that higher ED lactate values are associated with greater mortality in adults over 65 years of age, with or without the presence of infection.^9^ It has also been shown that there is a higher mortality with increasing age in patients with lactic acidosis.^9^ Therefore, in this study we hypothesized that older patients within the same lactate threshold level would have higher mortality rates, regardless of ED diagnosis. Finally, we hypothesized that additional lactate groupings (six) would provide greater mortality rate discrimination than the three traditionally used groupings for “low,” “intermediate,”, and “high.”

Hence, the primary objective of this study was to examine the combination of lactate level and age as predictors of mortality in adult patients who had a lactate level drawn in the ED as part of their initial work-up. Secondary objectives were 1) to compare traditional vs expanded lactate thresholds as predictors of mortality in three separate age cohorts; and 2) to identify the most common ED clinical impressions associated with an elevated lactate level.

## METHODS

### Study Design

This was a retrospective, observational, cohort study of adult patients (aged 18 years or older) who presented to the ED at an urban, tertiary-care teaching hospital from October 2009– May 2013. The average annual census for the ED is approximately 60,000 visits. The patients included were identified via Epic (Epic Systems, Verona, WI), the electronic health record for the hospital. The study is reported in accordance to the STROBE guidelines.^25^ It was reviewed by the institutional review board (the University of Kansas Medical Center Human Subjects Committee) and a waiver of informed consent was granted.

**Population Health Research Capsule**

What do we already know about this issue?

Lactate levels are a predictor of mortality among emergency department (ED) patients.

What was the research question?

This study investigates the effect of age across lactate levels as a predictor of mortality in ED patients.

What was the major finding of the study?

Increases in lactate or age, individually or in combination, were significantly associated with an increasing mortality risk.

How does this improve population health?

These results suggest that lactate levels and age together can be used to guide clinical practice, and traditional lactate thresholds may need to be both expanded and adjusted for age.

**Figure 1:**
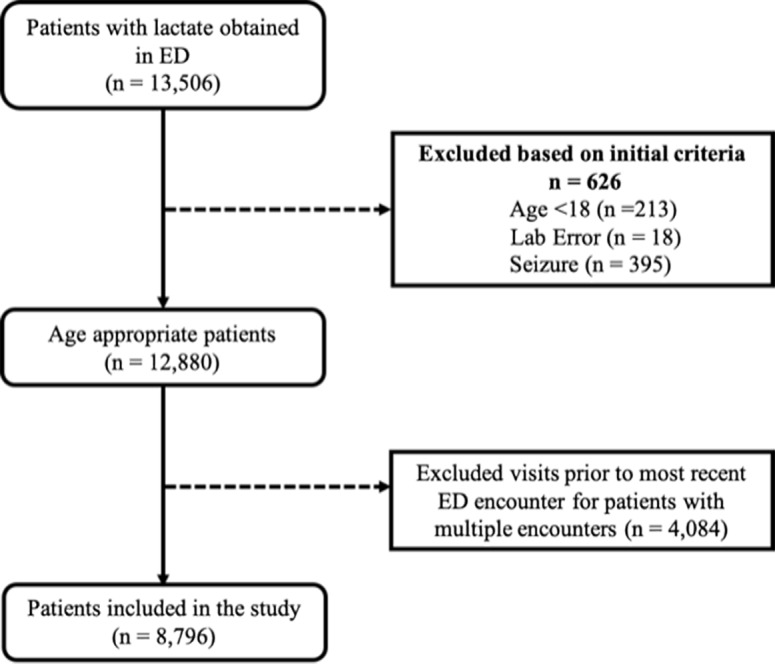
Study flowchart depicting the inclusion and exclusion criteria for ED patients with lactate levels. *ED*, emergency department.

Adult patients (aged 18 years or older) who had a lactate level drawn in the ED were included in the study. The ED in which this study was performed obtained a lactate level on individuals at the discretion of the emergency provider. At our institution, a sepsis protocol has existed since 2005, based upon recommendations from the Surviving Sepsis Campaign.^26^ This protocol educated all emergency providers on practice patterns and early recognition of sepsis, including the early obtainment of lactate levels. However, it was ultimately up to the emergency provider to determine whether or not a lactate level was necessary, and often lactate levels were ordered for many reasons other than suspected sepsis.^2^ Lactate levels were measured at the bedside with the Abbott point-of-care I-STAT (43%) (Abbott Laboratories, Chicago, IL), and in the hospital laboratory using a Beckman Coulter instrument (57%) (Beckman Coulter, Inc. Brea, CA). Bedside point-of-care lactate measurements have been shown to have excellent correlation with lab-reported lactate levels.^27^ We included only the first lactate level and clinical impression obtained during any ED visit in the dataset, and for individuals with multiple ED visits during the study period, only the first lactate level and clinical impression of the most recent encounter was included. We excluded patients with a diagnosis of seizure because associated high lactate levels carry a very low mortality risk in that subset and prior lactate research studies have excluded patients with seizures^2^,^12^ (Figure 1).

Transfer patients from outside hospitals are directly admitted to in-patient services at our institution; thus, there were no transfer patients included in this cohort. Demographics, including age, gender, race, vital signs, and diagnosis codes were obtained from the hospital discharge database and linked to lactate levels. The diagnosis codes included for acute infection and acute organ dysfunction, in Table 1 and 2, were defined by Angus et al in 2001.^28^ The primary outcome measure was in-hospital mortality, defined as patients who were admitted and died during the same encounter. Secondary outcome measures included hospital admission and admission to the intensive care unit (Table 1).

### Statistical Methods

We calculated descriptive statistics for age, lactate levels, vital signs, admission rates, mortality rates, and diagnoses. The patients were stratified into three age cohorts determined a priori: 18–39 years; 40–64 years; and 65 years or older. Patients were stratified into one of six lactate level cohorts that were also determined a priori: less than 2.0 mmol/L; 2.0–2.9 mmol/L; 3.0–3.9 mmol/L; 4.0–4.9 mmol/L; 5.0–5.9 mmol/L; and 6.0 mmol/L or greater. We performed analysis using SAS software version 9.3 (SAS Institute, Inc., Cary, NC). Chi-squared and Kruskal-Wallis tests were performed to compare age cohorts, lactate levels, and diagnoses to demographics, vital signs, and outcomes. We used logistic regression to estimate mortality odds ratios. Logistic models predicting mortality included either age groups (18–39, 40–64 and ≥ 65) or lactate level groupings (< 2, 2–2.9, 3–3.9, 4–4.9, 5–5.9, and ≥ 6), or a combination of both age and lactate level groupings as predictors. Logistic model using < 2 lactate level as the reference was stratified for each age group. Similarly, logistic model using the 18–39 age group as the reference was stratified for each lactate level grouping. We reported all results using an alpha level of 0.05. When applicable, 95% confidence intervals and standard error of the mean (SEM) were reported.

## RESULTS

Lactate levels were obtained on 13,506 patients, or 6.17% of the total patients seen in the ED over a 4.5-year period. Of these, we excluded 4710: 18 had lab error lactate values; 213 were younger than 18 years old; 4084 had multiple ED encounters; and 395 had a diagnosis of seizure (Figure 1). Thus, a total of 8796 patients were included in our analysis. A total of 474 (5.4%) in-hospital deaths occurred. Mortality rates generally increased with increasing lactate level and age (Tables 1 and 2). As lactate and/or age rose, patients were noted to have increased incidence of hypotension, septic shock, hospital admission, and ICU admission (Tables 1 and 2, p <0.05).

**Figure 2:**
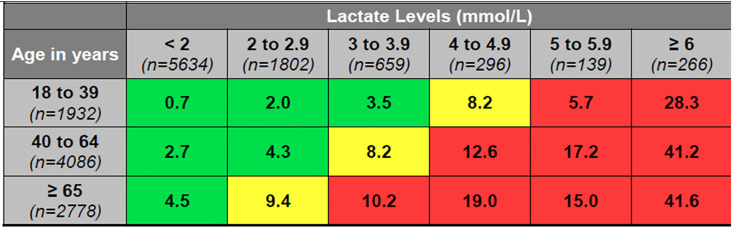
In-hospital mortality rate of ED patients by age and lactate level. *The mortality and lactate association within each age group row is significant with a p <0.0001. *mmol/L*, millimoles per liter; *ED*, emergency department.

Mortality generally increased within each age cohort with increasing lactate levels (Figure 2, p <0.0001). Figure 2 shows that patients with mean lactate levels less than 2.0 mmol/L had relatively low mortality rates in each defined age cohort: 0.7% in the 18–39 year old cohort; 2.7% in the 40–64 year old cohort; and 4.5% in the 65 years and older cohort, respectively. Mortality rates were higher in each age cohort in patients with lactate levels of 4.0 mmol/L–4.9 mmol/L: 8.2% in the 18–39 year old cohort; 12.6% in the 40–64 year old cohort; and 19.0% in the 65 years and older cohort (Figure 2). Mortality rates continued to increase, and at 6.0 mmol/L higher mortality rates were observed across all age cohorts: 28.3% in the 18–39 year old cohort; 41.2% in the 40–64 year old cohort; and 41.6% in the 65 years and older cohort (Figure 2, all p values <0.0001).

**Figure 3:**
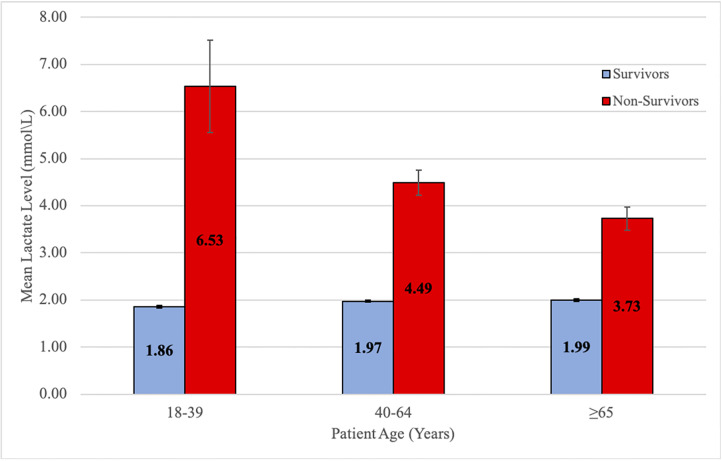
ED mean lactate level of survivors and non-survivors. *mmol/L*, millimoles per liter, *ED*, emergency department.

For adults aged 18–39 years, a lactate level of 4.0 mmol/L or higher was associated with a mortality of 5% or greater; for adults aged 40–64 years, this threshold decreased to ≥ 3.0 mmol/L; for adults aged 65 years or older, a lactate level of ≥ 2.0 mmol/L was associated with a 5% or greater mortality rate (Figure 2, p <0.0001). Mean lactate levels were consistently higher within each age cohort in non-survivors as compared to survivors, and mean lactate levels in non-survivors decreased as age increased (Figure 3, p <0.0001).

The mean lactate level of non-survivors was 6.5 mmol/L (SEM *=* 0.98) in the 18–39 year-old cohort, 4.5 mmol/L (*SEM =* 0.26) in the 40–64 year-old cohort, and 3.7 mmol/L (SEM *=* 0.24) in the 65 years or older cohort (p <0.0001). Mean lactate levels of survivors appeared consistent across the three age cohorts at approximately 2.0 mmol/L (SEM *=* 0.03, 0.02, 0.03, respectively). Mean lactate levels were different both overall and within the three age cohorts for gender (male vs female). Overall, males had a mean lactate of 2.22 as compared to 1.95 for females (p <0.0001).

Logistic modeling showed that both age and lactate were significant predictors of mortality. The odds ratios of mortality showed the same trend as the raw mortality rates in Figure 2: they generally increased with increasing lactate within each age cohort and with increasing age within each lactate-level cohort. When controlled for age, an increasing lactate level was still significantly associated with the outcome of in-hospital mortality (Appendix).

Outside of cardiac arrest, the primary clinical impressions resulting in the highest lactate levels were substance abuse, sepsis, and gastrointestinal (GI) bleed (Table 3). GI bleed had the highest ICU admission rate at 42.9%, followed by sepsis at 40.5%. While the most frequent clinical impression was abdominal pain, it had the second lowest mortality rate behind substance abuse and alcohol intoxication. Cardiac arrest had the highest mortality rate but only a marginal ICU admission rate, likely because patients were deceased prior to admission. Cardiac arrest, sepsis, GI bleed, respiratory distress, and pneumonia had the highest mortality rates while diabetic ketoacidosis (DKA), alcohol intoxication, and substance abuse were the clinical impressions associated with the lowest mortality rates.

## DISCUSSION

The use of lactate as a diagnostic indicator, prognostic marker, and/or resuscitation endpoint in patients with various disease states has been well described in the literature and has become routine in ED clinical practice.^11^,^15^,^23^,^29^–^35^ However, traditional lactate-level thresholds (low < 2.0 mmol/L; intermediate 2.0 to 3.9 mmol/L; and high ≥ 4.0 mmol/L) have been used to guide care without regard to patient age or underlying disease state.^11^ Expansion of these lactate-level thresholds and considerations of age and underlying disease states may prove useful in risk stratification and management decisions.

In our study, patients 65 years of age or older with lactate levels between 2.0–2.9 mmol/L had an in-hospital mortality rate of 9.4%. The 40–64 year-old cohort had a similar mortality rate (8.2%) with lactate levels between 3.0–3.9. The 18–39 year old cohort did not have a similar mortality rate (>8.2%) until their lactate levels exceeded 4.0 mmol/L, the level traditionally considered as “high.” The similar prognosis seen in varying lactate-level thresholds across age cohorts should raise caution in applying a simple “one size fits all” threshold approach to using lactate in clinical decisions.

It is important to address the individual and possible combined effects that age and lactate have on mortality, and what is driving and contributing to the increasing mortality rates noted in Figure 2. In an effort to examine this more closely, we used logistic regression modeling to evaluate the significance of lactate on mortality, using lactate <2 and age 18–39 separately and then combined as reference groups. Individually and within the age cohorts of 18–39, 40–64, and ≥ 65, lactate was found to be a significant predictor of mortality. Logistic regression modeling output, as noted in the appendix, supports the primary unadjusted findings of Figure 2. Either increases in lactate, age, or both, when compared to their respective reference cohort categorizations, does generally increase the odds of death. Using lactate levels clinically in the ED patient is complex and a provider should not be reassured by a seemingly “non-high (i.e., 2–3.9 mmol/L)” lactate level. Our results suggest that both variables, lactate and age, are important to consider in this patient population when assessing risk.

The existing literature evaluating lactate as a risk stratification tool for in-hospital mortality has predominantly looked at patients with the diagnosis of sepsis or trauma, raising the question of potential value of lactate use on the ED patient.^35^,^36^ Our study is the first in a large population to show that there is a clinically relevant difference in mortality across both expanded lactate cohorts and age cohorts. A statistical difference in lactate levels between genders was also noted; however, this finding was beyond the scope of this paper and could be explored in future research.

We observed a rise in mortality in each age cohort as lactate levels increased except for the 5.0–5.9 mmol/L lactate-level cohort, which actually had a slight decrease in mortality compared to the 4.0–4.9 mmol/L cohort in our 18–39 year-old and 65 years and older patients. This may be due to the lower number of patients (n = 139) in this lactate level cohort.

With the increasing number of lactate tests being ordered in EDs, it has become more important than ever for the clinician not to associate an elevated lactate solely with sepsis. It is well known that there are numerous causes for an elevated lactate and it is important to not narrow the differential diagnoses prematurely.^18^,^37^ Clinicians also need to be aware of diagnoses associated with high lactate levels but low mortality rates. Similar to the findings in our study (Table 3), DKA patients commonly present with elevated lactate levels and it has been shown that lactic acidosis in DKA is not associated with increased morbidity or mortality.^38^ Substance-abuse patients are another example of those who can have elevated lactate levels but low associated mortality rates, which was also consistent with our study (Table 3).^39^,^40^

## LIMITATIONS

This study, which analyzed a large number of patients within a single hospital, has several limitations. This was a retrospective analysis and carries the disadvantage of potential selection bias. Similar to Porter et al,^12^ the exclusion of multiple visits may overestimate the mortality rate; however, this avoids oversampling. Data abstracted for this study was from years 2009–2013; there could be more variation in practice patterns now. This study examined all patients with a lactate level measured in the ED, at the discretion of the treating provider and although protocols for sepsis screening are in place institutionally, it is difficult to extrapolate, retrospectively, a clinician’s rationale for deciding to order a lactate or not. Of note, the dataset did not capture specific causes for lab error and did not distinguish between arterial or venous samples; however, prior research has observed a strong correlation between arterial and venous concentrations.^41^

This data may not be generalizable to specific ED patients with presumed or known conditions and may vary depending on provider practice patterns and/or institutional guidelines. It is important to recognize that certain clinical disease processes and medications may also cause elevated lactate levels, and that a decision based upon a lactate level needs to be taken into context with the overall clinical picture. The clinical impressions used in this study were made by the treating emergency provider after the initial workup of the patient was completed, and there was no attempt to determine the etiology of the lactate levels associated with different clinical impressions and what effect the clinical impression may have. We only attempted to analyze associations, not causations. Additionally, patients may have had multiple clinical impressions associated with their encounter; however, only the primary clinical impression was ultimately included in this study.

Not all conditions that are associated with an elevated lactate level portend significant risk for mortality. We excluded patients with a diagnosis of seizure because this is a common ED presentation that is associated with an elevation of lactate but confers a known low risk of mortality.^2^,^12^ There may be other conditions associated with elevated lactate levels that also carry low risk for mortality that we did not exclude; these low-risk conditions have the potential to dilute the overall mortality risk shown in our results. On the contrary, including conditions such as cardiac arrest with an obvious high risk of death has the potential to overestimate the overall mortality risk in this study. Even if the included 65 patients with cardiac arrest (45 deaths) were removed, overall mortality in the study would only have dropped from 5.4% to 4.9%.

There may be significant differences in lactate prognostic ability based upon gender and race that could be explored in future research. Physiologic characteristics of lactate metabolism and clearance, such as body mass index, diet, and medication use, are confounding factors that could account for differences in lactate level and prognostic ability that could not be controlled for in this study.

## CONCLUSION

This study suggests that the combination of increasing lactate levels and/or age are associated with increasing in-hospital mortality. Our findings suggest that lactate levels may be used as a prognostic tool to help risk stratify ED patients. These findings suggest that the traditional lactate level thresholds currently used to guide clinical practice may need to be both expanded and adjusted for age. Clinicians need to be aware of the many potential causes of lactate elevation as the clinical and prognostic importance of an elevated lactate also varies widely by disease state.

## Supplementary Information



## Figures and Tables

**Table 1:** Population characteristics by lactate levels.

		Initial lactate level (mmol/L)						

		< 2 (N=5634)	2 – 2.9 (N=1802)	3 – 3.9 (N=659)	4 – 4.9 (N=296)	5 – 5.9 (N=139)	≥ 6 (N=266)	P-value
Demographics	Age (median, mean ± SD)	55, 54.3 (± 18.5)	57, 56.5 (± 18.0)	57, 56.8 (± 17.3)	56, 56 (± 18.0)	55, 54.6 (± 18.6)	57, 56.4 (± 17.7)	<0.0001*
	Male, *n* (%)	2,256 (45.4)	932 (51.7)	365 (55.4)	157 (53.0)	84 (60.4)	156 (58.6)	<0.0001
	White, *n* (%)	3,593 (63.8)	1147 (63.7)	423 (64.3)	186 (62.8)	76 (54.7)	148 (55.6)	0.02
Clinical variables	Lactate, mmol/L (median, mean ± SD)	1.3, 1.3 (± 0.4)	2.3, 2.4 (± 0.3)	3.4, 3.4 (± 0.3)	4.4, 4.4 (± 0.3)	5.3, 5.4 (± 0.3)	8.3, 9.4 (± 3.4)	<0.0001*
	SBP, mmHg (median, mean ± SD)	134, 135 (± 27)	132, 134 (± 29)	127, 130 (± 31)	125, 126 (± 31)	127, 126 (± 30)	121, 126 (± 36)	<0.0001*
	Hypotensive (SBP<90) , *n* (%)	171 (3.2)	82 (4.8)	47 (7.5)	33 (11.4)	14 (10.7)	33 (13.3)	<0.0001
	Sepsis, *n* (%)	327 (5.8)	187 (10.4)	101 (15.3)	57 (19.3)	24 (17.3)	33 (12.4)	<0.0001
	Severe sepsis, *n* (%)	141 (2.5)	123 (6.8)	65 (9.9)	45 (15.2)	20 (14.4)	29 (10.9)	<0.0001
	Septic shock, *n* (%)	44 (0.8)	31 (1.7)	18 (2.7)	19 (6.4)	9 (6.5)	20 (7.5)	<0.0001
	Acute infection, *n* (%)	1,456 (25.8)	471 (26.1)	179 (27.2)	75 (25.3)	31 (22.3)	45 (16.9)	0.03
	Acute organ dysfunction, *n* (%)	791 (14.0)	317 (17.6)	156 (23.7)	86 (29.1)	33 (23.7)	56 (21.1)	<0.0001
Outcome	Admitted, *n* (%)	3,858 (68.5)	1,420 (78.8)	567 (86.0)	274 (92.6)	124 (89.2)	237 (89.1)	<0.0001
	Admitted to ICU, *n* (%)	362 (6.4)	209 (11.6)	140 (21.2)	93 (31.4)	47 (33.8)	121 (45.5)	<0.0001
	Mortality, *n* (%)	157 (2.8)	100 (5.6)	53 (8.0)	41 (13.9)	19 (13.7)	104 (39.1)	<0.0001

* Based on Kruskal-Wallis (non-parametric) test.

*SBP*, Systolic Blood Pressure; *ICU*, Intensive Care Unit; *mmol/L*, millimoles per liter; *mmHg*, millimeters of mercury; *SD*, standard deviation.

**Table 2:** Population characteristics by age.

		Age			

		18 – 39 (N=1932)	40 – 64 (N=4086)	≥ 65 (N=2778)	P-value
Demographics	Age (median, mean ± SD)	30, 29.4 (± 6.1)	53, 53.1 (± 6.7)	75, 76 (± 8.1)	<0.0001*
	Male, n (%)	893 (46)	2,034 (50)	1,323 (48)	0.02
	White, n (%)	1,055 (54.7)	2,621 (64.2)	1,897 (68.3)	<0.0001
Clinical variables	Lactate, mmol/L (median, mean ± SD)	1.5, 2.0 (± 1.7)	1.6, 2.1 (± 1.7)	1.6, 2.1 (± 1.7)	<0.0001*
	SBP, mmHg (median, mean ± SD)	131, 132 (± 24)	133, 134 (± 29)	133, 135 (± 30)	0.09*
	Hypotensive (SBP<90) , n (%)	39 (2.1)	207 (5.3)	134 (5.1)	<0.0001
	Sepsis, n (%)	97 (5.0)	345 (8.4)	287 (10.3)	<0.0001
	Severe sepsis, n (%)	51 (2.6)	196 (4.8)	176 (6.3)	<0.0001
	Septic Shock, n (%)	13 (0.7)	73 (1.8)	55 (2.0)	<0.001
	Acute infection, n (%)	418 (21.6)	1,002 (24.5)	834 (30.1)	<0.0001
	Acute organ dysfunction, n (%)	148 (7.7)	670 (16.4)	621 (22.4)	<0.0001
Outcome	Admitted, n (%)	1,132 (58.6)	3,002 (73.5)	2,346 (84.5)	<0.0001
	Admitted to ICU, n (%)	164 (8.5)	444 (10.9)	364 (13.1)	<0.0001
	Mortality, n (%)	40 (2.1)	216 (5.3)	218 (7.9)	<0.0001

* Based on Kruskal-Wallis (non-parametric) test.

*SBP*, Systolic Blood Pressure; *ICU*, Intensive Care Unit; *mmol/L*, millimoles per liter; *mmHg*, millimeters of mercury; *SD*, standard deviation.

**Table 3:** Top clinical impressions of lactate >2 mmol/L by frequency.

Clinical impression	Frequency	Lactate (Mean)	Age (Mean)	Admitted ICU (%)	Mortality (%)
Abdominal pain	264	2.8	51.9	6.1	3.0
Pneumonia	228	3.1	63.7	20.6	12.3
Respiratory distress	200	3.5	59.5	32.5	14.5
Sepsis	190	4.3	58.4	40.5	21.1
UTI	152	2.9	62.5	5.3	3.3
AMS	142	3.9	57.1	21.1	7.0
N/V/D	120	3.2	53.6	4.2	5.0
Fever	102	2.7	54.8	5.9	5.9
DKA	99	3.5	45.7	23.2	1.0
Dehydration	79	3.1	60.5	8.9	3.8
GI bleed	70	4.0	59.6	42.9	15.7
Cellulitis	68	2.8	50.6	4.4	4.4
Cardiac arrest	65	10.5	62.2	43.1	70.8
Liver failure	52	3.2	54.4	17.3	11.5
Infectious abdominal diseases	48	3.9	57.7	8.3	6.3
Alcohol intoxication	38	3.3	51.1	10.5	0.0
Substance abuse*	35	4.4	38.0	17.1	0.0

* Includes PCP, cocaine, and unknown ingestion.

*ICU*, intensive care unit; *UTI*, urinary tract infection; *AMS*, altered mental status; *N/V/D*, nausea/vomitting/diarrhea; *DKA*, diabetic ketoacidosis; *GI*, gastrointestinal; *mmol/L*, millimoles per liter.
