# Supplementary material for: Age-adjusted and Expanded Lactate Thresholds as Predictors of All-Cause Mortality in the Emergency Department
Source: West J Emerg Med. 2020 Aug 20;21(5):1249–57. doi: 10.5811/westjem.2020.5.46811 (PMC7514398; doi:10.5811/westjem.2020.5.46811)
Supplement: Supplementary file 1 [file wjem-21-1249-s001.docx]

**Appendix A.** Mortality odds ratios of ED patients by lactate level within age group.*

|  | **Lactate Levels (mmol/L)** | | | | | |
| --- | --- | --- | --- | --- | --- | --- |
| **Age in Years** | **< 2**  *(n=5634)* | **2 to 2.9**  *(n=1802)* | **3 to 3.9**  *(n-659)* | **4 to 4.9**  *(n=296)* | **5 to 5.9**  *(n=139)* | **≥ 6**  *(n=266)* |
| **18 to 39**  *(n=1932)* | **Reference**  **Group** | **2.98**  *(1.10-8.05)* | **5.32**  *(1.61 – 17.54)* | **13.06**  *(4.24 – 40.23)* | **8.86**  *(1.84 – 42.62)* | **57.60**  *(23.02 – 144.17)* |
| **40 to 64**  *(n=4086)* | **Reference**  **Group** | **1.62**  *(1.08 – 2.43)* | **3.20**  *(2.02 – 5.06)* | **5.15**  *(2.94 – 9.03)* | **7.42**  *(3.72 – 14.82)* | **25.08**  *(16.46 – 38.21)* |
| **≥ 65**  *(n=2778)* | **Reference**  **Group** | **2.20**  *(1.54 – 3.14)* | **2.41**  *(1.47 – 3.96)* | **4.99**  *(2.88 – 8.63)* | **3.75**  *(1.53 – 9.20)* | **15.13**  *(9.37 -24.4)* |

*Odds Ratios (95% Confidence Intervals) within each age grouping compared to patients with a lactate <2

*ED,* emergency department; *mmol,* millimoles; *L,* liter.

**Appendix B:** Mortality odds ratios of ED patients by age within each lactate level group.*

|  | **Lactate Levels (mmol/L)** | | | | | |
| --- | --- | --- | --- | --- | --- | --- |
| **Age in Years** | **< 2**  *(n=5634)* | **2 to 2.9**  *(n=1802)* | **3 to 3.9**  *(n-659)* | **4 to 4.9**  *(n=296)* | **5 to 5.9**  *(n=139)* | **≥ 6**  *(n=266)* |
| **18 to 39**  *(n=1932)* | **Reference**  **Group** | **Reference**  **Group** | **Reference**  **Group** | **Reference**  **Group** | **Reference**  **Group** | **Reference**  **Group** |
| **40 to 64**  *(n=4086)* | **4.09**  *(2.04 – 8.21)* | **2.23**  *(0.98 – 5.04)* | **2.46**  *(0.84 – 7.18)* | **1.61**  *(0.57 – 4.60)* | **3.42**  *(0.71 – 16.42)* | **1.78**  *(0.86 – 3.69)* |
| **≥ 65**  *(n=2778)* | **6.88**  *(3.44 – 13.77)* | **5.09**  *(2.29 – 11.29)* | **3.12**  *(1.05 – 9.28)* | **2.63**  *(0.93 – 7.45)* | **2.91**  *(0.55 – 15.46)* | **1.81**  *(0.84 -3.89)* |

*Odds Ratios (95% Confidence Intervals) within each Lactate grouping compared to patients with an Age 18 to 39.

*ED,* emergency department; *mmol,* millimoles; *L,* liter.

**Appendix C.** Mortality odds ratios of ED patients by each age and lactate level group*

|  | **Lactate Levels (mmol/L)** | | | | | |
| --- | --- | --- | --- | --- | --- | --- |
| **Age in Years** | **< 2**  *(n=5634)* | **2 to 2.9**  *(n=1802)* | **3 to 3.9**  *(n-659)* | **4 to 4.9**  *(n=296)* | **5 to 5.9**  *(n=139)* | **≥ 6**  *(n=266)* |
| **18 to 39**  *(n=1932)* | **Reference**  **Group** | **2.98**  *(1.10 – 8.05)* | **5.32**  *(1.61 – 17.54)* | **13.06**  *(4.24 – 40.23)* | **8.86**  *(1.84 – 42.62)* | **57.60**  *(23.02 – 144.17)* |
| **40 to 64**  *(n=4086)* | **4.09**  *(2.04 – 8.21)* | **6.62**  *(3.18 – 13.79)* | **13.07**  *(6.09 – 28.08)* | **21.07**  *(9.19 – 48.29)* | **30.35**  *(12.06 – 76.36)* | **102.55**  *(48.82 – 215.39)* |
| **≥ 65**  *(n=2778)* | **6.88**  *(3.44 – 13.77)* | **15.14**  *(7.43 – 30.82)* | **16.58**  *(7.53 – 36.54)* | **34.30**  *(15.04 – 78.21)* | **25.80**  *(8.70 – 76.57)* | **104.04**  *(47.72 -226.83)* |

*Odds Ratios (95% Confidence Intervals) within each grouping compared to patients with an Age 18 to 39 with Lactate < 2

*ED,* emergency department; *mmol,* millimoles; *L,* liter.
